# Case Analysis of Neonatal Acute Liver Failure Presumably Caused by Gestational Alloimmune Liver Disease

**DOI:** 10.1055/a-2950-1681

**Published:** 2026-09-22

**Authors:** Qingying Wang, Jingyi Zhang, Yi Xu, Xia Chen, Fang Huang, Jingjing Wu, Yongchao Jiang, Gaoyan Chen

**Affiliations:** 1 Department of Pediatrics 74731 Xiangyang Central Hospital Graduate Joint Training Base, School of Medicine, Wuhan University of Science and Technology; 2 Department of PediatricsXiangyang Central Hospital, Affiliated Hospital of Hubei University of Arts and Science, Clinical Research Center for Pediatric Diseases and Rare DiseasesXiangyangHubeiPeople’s Republic of China

**Keywords:** neonatal acute liver failure, gestational alloimmune liver disease, exchange transfusion, coagulopathy, cholestasis

## Abstract

Neonatal acute liver failure is a rare, life-threatening condition. We present a 33 ^6/7^ -week-old neonate with severe coagulopathy and hyperbilirubinemia refractory to vitamin K _1_ , plasma, and platelet transfusions. The mother had a history of two previous neonatal deaths, one of whom had similar coagulopathy. Gestational alloimmune liver disease (GALD) was strongly suspected following multidisciplinary review, although a definitive diagnosis could not be confirmed because of negative labial salivary gland iron staining and the decision to decline liver biopsy. Double-volume exchange transfusion on day 15 rapidly improved bilirubin and coagulation parameters, allowing discharge in stable condition. Mild recurrent cholestasis occurred after discharge. This case underscores that refractory coagulopathy and cholestasis unresponsive to conventional therapy, especially in the presence of a relevant family history, should raise suspicion for immune-mediated causes such as GALD. Exchange transfusion may be critical for improving outcomes.

##

**Key Points**

- Refractory coagulopathy in a neonate with relevant family history.
- GALD strongly suspected despite inconclusive diagnostics.
- Exchange transfusion resulted in rapid clinical improvement.
- This study highlights the importance of considering immune-mediated causes in neonatal liver failure.

## Introduction

Neonatal acute liver failure (NALF) is a severe hepatic disorder occurring in the neonatal period, characterized by rapid deterioration of liver function and loss of metabolic and detoxification capabilities. Despite its low clinical incidence, NALF progresses rapidly with high mortality, posing a significant management challenge in the neonatal intensive care unit (NICU). 1 Its etiology is complex and diverse, with known causes, including gestational alloimmune liver disease (GALD), viral infections (such as cytomegalovirus), hemophagocytic lymphohistiocytosis, and other rare causes; however, nearly half of cases remain unexplained. 2

GALD, caused by maternal antibodies attacking fetal hepatocytes, is the most common cause of unexplained neonatal liver failure. Treatment typically involves exchange transfusion and intravenous immunoglobulin. 3

This report describes a preterm infant at 33 ^6/7^ weeks of gestation with a clear positive family history. The patient presented immediately after birth with severe coagulation dysfunction and cholestasis that were refractory to conventional treatment, leading to a complex diagnostic and therapeutic journey. Through this case, we aim to explore clinical differential diagnostic approaches for such rare refractory coagulation disorders, emphasizing the pivotal role of exchange transfusion therapy in their management. This study seeks to provide insights into the clinical management of similar complex cases.

## Clinical Information

### General Information and Birth History

A male infant was delivered by cesarean section at 33 ^6/7^ weeks of gestation to a Gravida 3 Para 3 (G3P3) mother due to oligohydramnios and a significant obstetric history. Birth weight was 2150 g, with Apgar scores of 8–9. The infant was transferred to our NICU at 17:00 on the same day due to “respiratory distress, preterm birth, and high-risk status.” The mother had gestational diabetes mellitus, and a prenatal ultrasonogram had noted a crossing of the left and right pulmonary arteries.

Both of the infant’s older brothers had adverse birth outcomes: The first died of unknown causes during the neonatal period; the second was admitted to the NICU for meconium-stained amniotic fluid, diagnosed with patent ductus arteriosus and pulmonary hemorrhage, and died despite mechanical ventilation and transfusions of blood products for refractory coagulopathy.

### Admission Presentation and Physical Examination

**Fig. 1:**
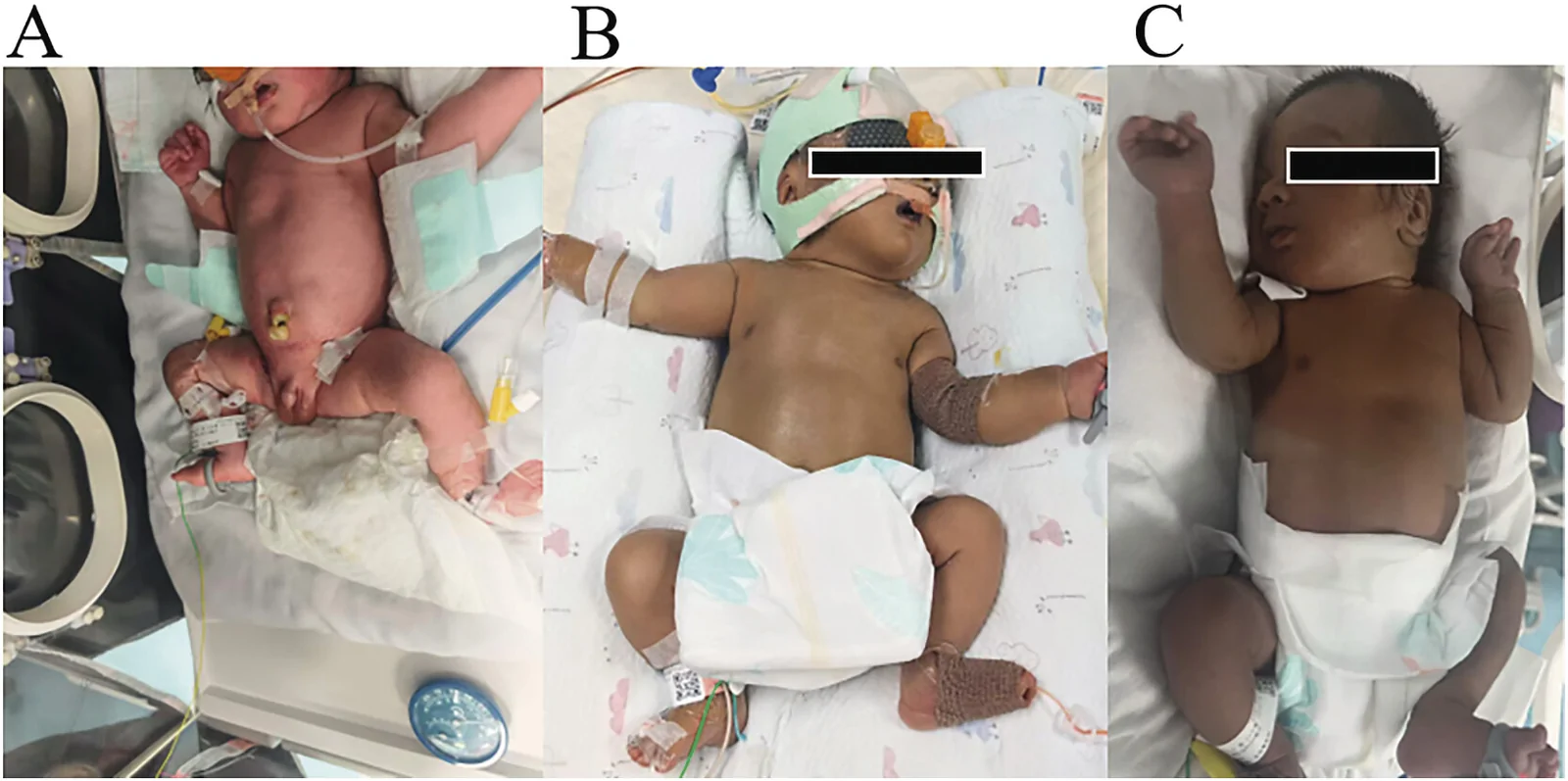
( **A** ) Photograph of the patient upon admission. ( **B** ) Photograph of the patient prior to exchange transfusion therapy. ( **C** ) Photograph of the patient upon discharge.

While breathing room air, the infant had labored breathing with mild retractions and an absence of cyanosis. Physical examination revealed mild jaundice of the skin and mucous membranes; besides, scattered petechiae and ecchymoses were noted on the skin of both lower limbs and the trunk ( Fig. 1 ).

### Supportive Tests

**Fig. 2:**
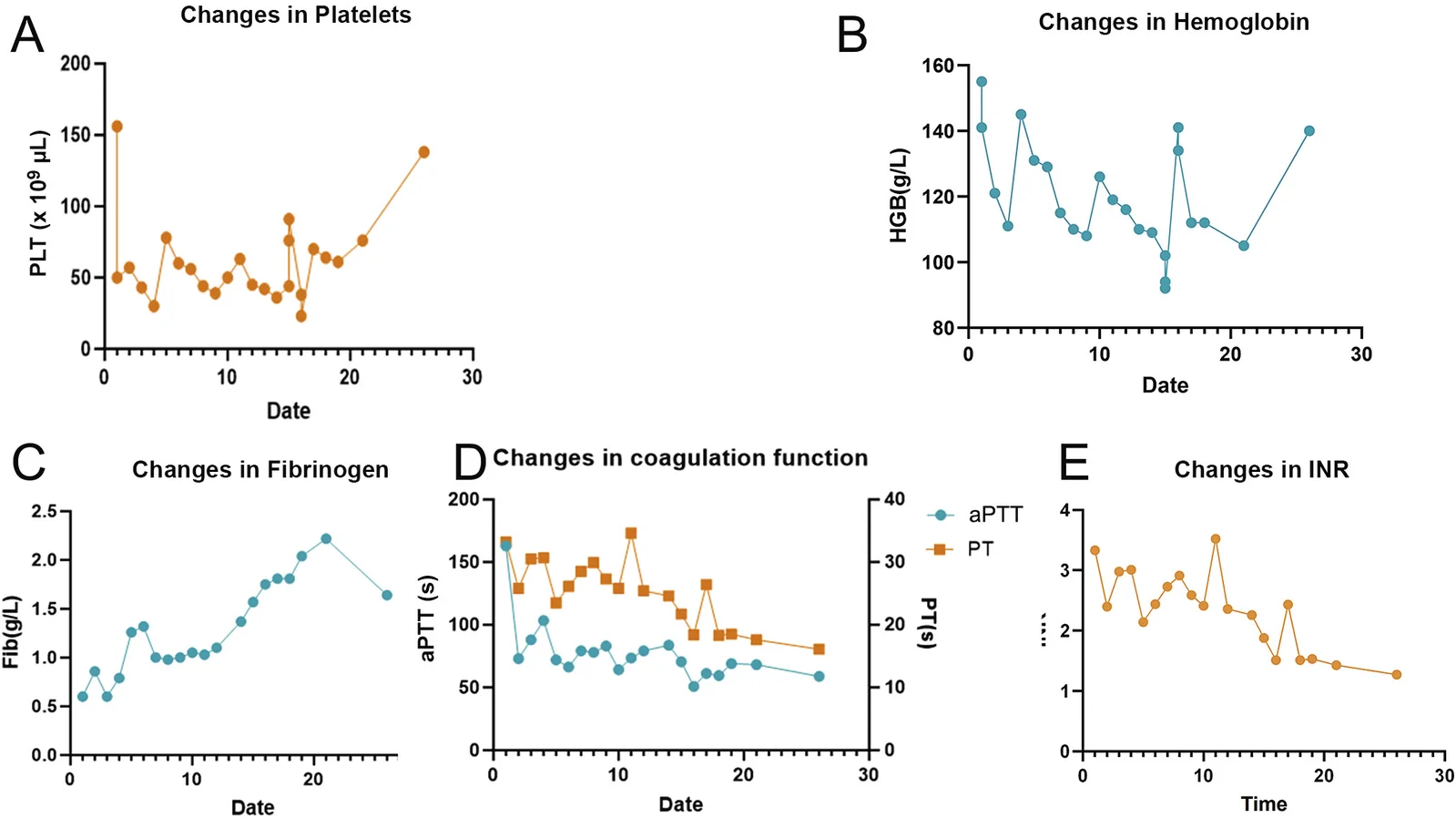
( **A** ) Platelet changes. ( **B** ) Hemoglobin changes. ( **C** ) Fibrinogen changes. ( **D** ) aPTT and PT changes. ( **E** ) INR changes. aPTT, activated partial thromboplastin time; Fib, fibrinogen; INR, International Normalized Ratio; PT, prothrombin time.

Complete blood count and coagulation studies revealed reductions in red cells, platelets, and multiple coagulation parameters ( Fig. 2 ). Repeated administration of plasma, platelets, and red blood cells yielded poor therapeutic response. Coagulation factor activity analysis revealed that the activities of other coagulation factors in the intrinsic, extrinsic, and common pathways were severely depressed early in the disease course, except for normal Factor VIII (FVIII) activity. Although some improvement was observed following therapeutic interventions, factor activities remained below the normal range.

#### Liver Function Tests

**Fig. 3:**
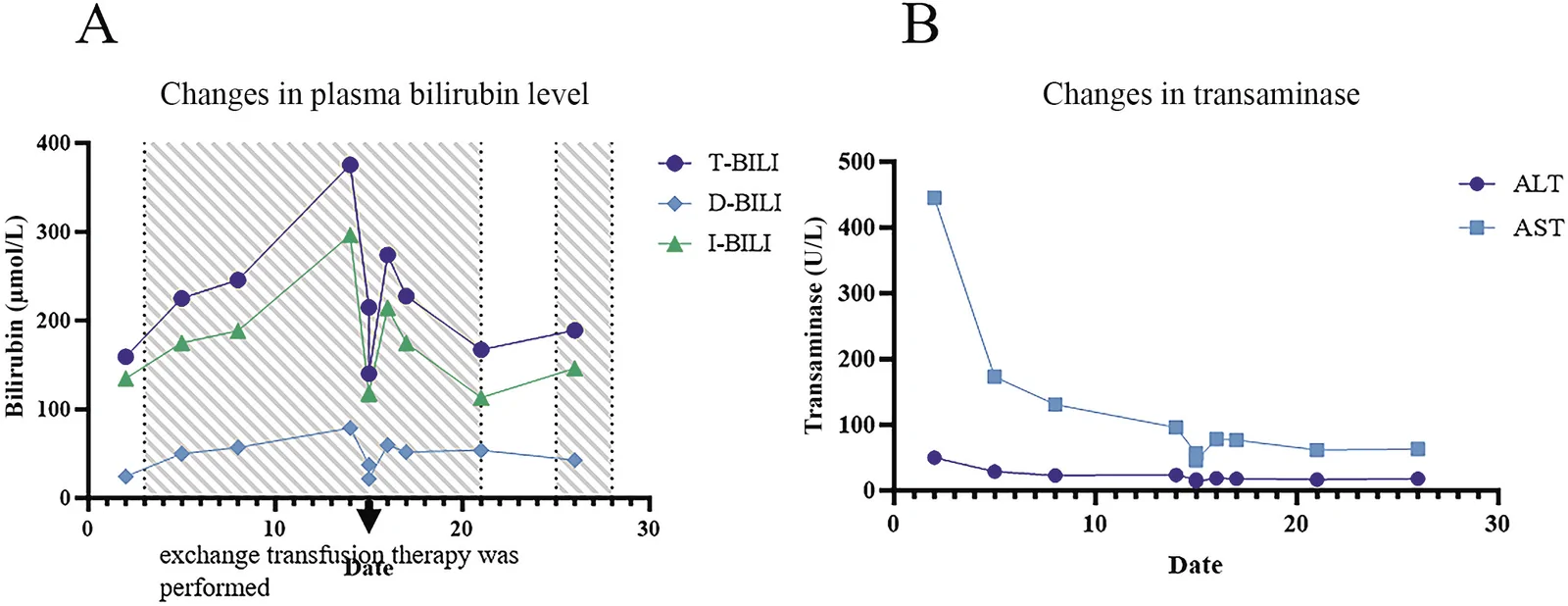
( **A** ) Changes in bilirubin levels. Areas shaded in gray indicate blue light therapy. As shown, the patient underwent an exchange transfusion on the 15th day of hospitalization. ( **B** ) Changes in liver transaminase levels.

Despite blue light therapy after admission, bilirubin levels remained elevated while liver transaminase levels decreased ( Fig. 3 ). Bile enzyme dissociation was observed on day 15 of hospitalization. Following exchange transfusion, liver function parameters gradually improved.

#### Immunological Tests

Evaluation of the anticoagulant system revealed marked reductions in plasma protein C activity (11.0%) and protein S level (14.3%). Both antigen and activity of von Willebrand factor (VWF) were markedly elevated at 532.0% and 483.0%, respectively. Factor XIII antigen was 36.1%, indicating a decrease. The extractable nuclear antigen–antibody panel showed a mild elevation in the anti-Ro-52 antibody (33.6 RU/mL). ADAMTS13 (a disintegrin and metalloproteinase with a thrombospondin type 1 motif, member 13) activity was within the normal range at 79.6%, and no ADAMTS13 inhibitor was detected.

#### Genetic Testing

To elucidate the etiology, genetic analysis interrogating the nuclear genome was performed. Results revealed a heterozygous variant c.3223-80A>G in the VWF gene (NM_000552.5), detected using single nucleotide variant and small insertion/deletion sequencing technology. According to the American College of Medical Genetics and Genomics (ACMG) guidelines, this variant was classified as a variant of uncertain significance (VUS, PM2_Supporting+PP3). Concurrent analyses of copy number variants, loss of heterozygosity, structural variants, mitochondrial genome variants, and gene-specific short tandem repeat (STR) expansions failed to identify any pathogenic variants accounting for the patient’s clinical phenotype. The VWF gene is associated with von Willebrand disease, and its abnormalities can lead to bleeding tendencies. However, considering the patient’s clinical presentation and family history, there is insufficient evidence to establish a definitive pathogenic association between this variant and the observed phenotype.

#### Additional Investigations

No clinically significant ascites or splenomegaly was identified during the initial hospitalization. Blood glucose levels remained within the normal range with routine clinical management, and no documented episodes of hypoglycemia were observed during hospitalization.

Severe iron metabolism abnormalities were observed, with markedly elevated ferritin levels (3808.00 ng/mL) alongside reduced total iron-binding capacity (28.61 μmol/L) and transferrin (0.91 g/L), consistent with iron overload. Magnetic resonance imaging (MRI) of the liver and pancreas revealed no morphological changes or abnormal signals. A labial salivary gland biopsy showed no histological abnormalities, and a negative iron stain ruled out the possibility of localized iron deposition.

### Clinical Course

#### Initial Management and Disease Progression (Days 1–8)

Upon admission, the patient received antimicrobial therapy, respiratory support, and nutritional supplementation, along with phototherapy for hyperbilirubinemia. Coagulopathy showed no significant improvement despite daily vitamin K _1_ (10 mg) and aggressive transfusions of blood products.

On day 2, the patient experienced sudden massive gastrointestinal hemorrhage with hemorrhagic shock. Endotracheal intubation and vasoactive drug support were performed. Vital signs stabilized following aggressive blood transfusion and symptomatic hemostasis. Given the patient’s coagulation dysfunction, active bleeding, and positive family history of coagulation disorders, a clinical suspicion of a hereditary disease arose. Consequently, protein C/S testing was performed for the patient and both parents, and whole-exome sequencing along with a coagulation/bleeding disorder-related gene panel were initiated to determine the etiology. During this phase, the patient’s jaundice progressively worsened, and the skin developed a dull, dark hue.

#### Diagnostic Exploration and Multidisciplinary Team Consultation (Days 9–13)

Due to poor response to initial therapy, multiple multidisciplinary team (MDT) consultations were organized. The first MDT (Day 9), informed by the returned protein C and S levels, ruled out their inherited deficiencies and narrowed the differential diagnosis to entities such as thrombotic microangiopathy and hemophagocytic lymphohistiocytosis. During the second MDT consultation (Day 13), the patient showed no active bleeding but persisted with jaundice, edema, and tea-colored urine. Combining the test results (e.g., normal ADAMTS13 activity), the panel ruled out classic thrombotic thrombocytopenic purpura. Given the maternal pregnancy history, cholestasis, and coagulation abnormalities, GALD remained the leading diagnostic consideration.

However, definitive diagnosis was not achieved due to negative imaging studies, negative iron staining in lip gland biopsy, and absence of pathogenic mutations in genetic testing.

#### Exchange Transfusion and Recovery (Days 15–29)

On day 15, total bilirubin (TB) rose to 375.4 μmol/L, meeting the criterion for exchange transfusion. Double-volume exchange transfusion was performed. Postexchange, bilirubin levels decreased markedly, accompanied by progressive improvement in coagulation function, including decreased International Normalized Ratio (INR) and shortened prothrombin time and activated partial thromboplastin time, as well as increased fibrinogen levels, and an increased fibrinogen levels.

A transient fever occurred postexchange, and then resolved with antimicrobial therapy. Subsequently, the patient’s jaundice gradually subsided, coagulation function remained stable, and the condition entered the recovery phase.

### Outcome and Follow-up

After discharge, the patient was maintained on oral vitamin K _1_ , vitamins A and D, and ursodeoxycholic acid, with regular follow-up. On September 3, 2025, the patient was readmitted due to recurrent jaundice (total bilirubin (TB) 115.4 μmol/L, direct bilirubin (DB) 47 μmol/L), which improved with phototherapy.

Subsequent follow-ups (September 17 and September 30, 2025) showed fluctuating increases in direct bilirubin levels (52.7 μmol/L → 72.8 μmol/L), indicating recurrent cholestasis, though coagulation function remained stable. Progressive hepatomegaly and splenomegaly were also observed during follow-up evaluations. During this period, the patient’s growth and development parameters were consistent with those of age-matched peers. Long-term nutritional monitoring and follow-up will be required in the future.

## Discussion

NALF is a rare, life-threatening condition characterized by complete or near-complete loss of liver function during the neonatal period. Primary causes include galactosemia (GALD), viral infections, hematologic disorders, metabolic diseases, and ischemic injury; however, the etiology remains unknown in most cases. 4 5 The clinical manifestations of NALF are diverse, including feeding refusal, growth arrest, hypoglycemia, vitamin K-resistant bleeding, and cholestatic jaundice. 6

This case involves a preterm infant who presented with severe coagulation disorders and hyperbilirubinemia immediately after birth, with poor response to vitamin K _1_ administration and plasma transfusion. The mother had a history of two previous fetal or early neonatal deaths, one of which was also associated with coagulation abnormalities. Given the significant obstetric history and characteristic clinical features, GALD was considered the leading diagnostic consideration. Differential diagnostic evaluations for NALF were performed during hospitalization. Clinical manifestations, laboratory examinations, imaging findings, and whole exome sequencing did not reveal evidence strongly suggestive of inherited metabolic disorders such as tyrosinemia or galactosemia. In addition, no clinical or laboratory evidence suggested congenital viral infection, including TORCH (Toxoplasmosis, Other infections, Rubella, Cytomegalovirus, and Herpes simplex virus)-related diseases, cytomegalovirus infection, or herpes simplex virus infection. Collectively, these findings made metabolic and infectious etiologies less likely and further supported the clinical suspicion of GALD.

GALD is an alloimmune-mediated disorder in which maternal antibodies cross the placenta and target fetal hepatocytes, representing a significant cause of NALF. 2 Typical clinical manifestations include early-onset cholestasis, severe coagulation disorders, hypofibrinogenemia, and, in some cases, accompanied by extrahepatic iron deposition. 7 8 In this case, the patient exhibited early-onset coagulation disorders, cholestasis, markedly elevated ferritin levels, poor response to conventional therapy, and rapid clinical improvement following exchange transfusion, all of which were consistent with the clinical features of GALD. However, a definitive diagnosis could not be established due to several key diagnostic evaluations being unavailable, including the negative Perls’ iron stain of the labial salivary gland, the lack of a liver biopsy, and the unavailability of maternal antibody testing. In addition, serum ammonia was not assessed during the initial hospitalization, which represents another limitation of the present report. Despite these limitations, the overall clinical presentation, obstetric history, laboratory findings, and therapeutic response collectively supported GALD as the most likely diagnosis.

It should be noted that negative extrahepatic iron staining does not completely rule out GALD, as both the sampling site and disease stage can influence results. 9 With no evidence of extrahepatic iron deposition, liver biopsy with C5b-9 immunohistochemical staining was considered; a positive result would suggest GALD. 10 11 Unfortunately, a definitive diagnosis remains elusive as the family declined liver biopsy.

Genetic testing identified a VWF c.3223–80A>G variant classified as a VUS, which was inconsistent with the clinical phenotype. This highlights limitations of genetic testing in rare disease diagnosis: First, existing panels inadequately cover immune-mediated disorders; second, clinical interpretation of VUS relies on database accumulation and functional validation, making definitive confirmation challenging in the short term. 12 Therefore, genetic testing should be used as an adjunct diagnostic tool in this case, the results of which must be interpreted in conjunction with clinical and immunological findings.

In the present case, rapid improvement in coagulation function, normalization of INR, and reduction in bilirubin levels following exchange transfusion supported its potential therapeutic benefit in suspected GALD. Exchange transfusion not only removes potentially pathogenic antibodies and inflammatory factors from the circulation but also replenishes coagulation factors and improves hepatic microcirculation. 13 14 Previous studies have also confirmed that early exchange transfusion combined with intravenous immunoglobulin therapy may significantly reduce GALD-related mortality. 9 In this patient, only one session of double-volume exchange transfusion was performed because coagulation function and bilirubin levels improved rapidly after treatment, and no further active bleeding was observed. Therefore, additional exchange transfusion was not considered clinically necessary during the acute phase.

Notably, although this patient demonstrated good recovery of coagulation function, cholestasis recurred postdischarge. This suggests that while exchange transfusion may improve acute liver injury, it cannot fully correct subsequent bile excretion disorders. Clinically, long-term follow-up with dynamic monitoring of liver function and bile metabolism is essential in similar patients.

## Conclusion

This case highlights the clinical complexity and diagnostic challenges of GALD-like disorders. For refractory coagulation disorders with cholestasis in neonates unresponsive to conventional therapy, a family history of clustering should prompt consideration of immune-mediated liver injury. Early implementation of empirical exchange transfusion or combined immunoglobulin therapy is warranted. A systematic diagnostic approach and dynamic assessment can enhance the recognition and survival rates of such rare diseases.
